# Unraveling structural and compositional information in 3D FinFET electronic devices

**DOI:** 10.1038/s41598-019-48117-0

**Published:** 2019-08-12

**Authors:** Henrique Trombini, Gabriel Guterres Marmitt, Igor Alencar, Daniel Lorscheitter Baptista, Shay Reboh, Frédéric Mazen, Rafael Bortolin Pinheiro, Dario Ferreira Sanchez, Carlos Alberto Senna, Bráulio Soares Archanjo, Carlos Alberto Achete, Pedro Luis Grande

**Affiliations:** 1 Ion Implantation Laboratory, Institute of Physics, Federal University of Rio Grande do Sul, CEP, 91501-970 Porto Alegre, Brazil; 2 CEA-LETI, MINATEC Campus, F-38054 Grenoble, France; 3 Paul Scherrer Instituit, 5232 Villigen, Switzerland; 4 National Institute of Metrology, Quality and Technology, CEP, 25250-020 Duque de Caxias, Brazil

**Keywords:** Characterization and analytical techniques, Electronic devices

## Abstract

Non-planar Fin Field Effect Transistors (FinFET) are already present in modern devices. The evolution from the well-established 2D planar technology to the design of 3D nanostructures rose new fabrication processes, but a technique capable of full characterization, particularly their dopant distribution, in a representative (high statistics) way is still lacking. Here we propose a methodology based on Medium Energy Ion Scattering (MEIS) to address this query, allowing structural and compositional quantification of advanced 3D FinFET devices with nanometer spatial resolution. When ions are backscattered, their energy losses unfold the chemistry of the different 3D compounds present in the structure. The FinFET periodicity generates oscillatory features as a function of backscattered ion energy and, in fact, these features allow a complete description of the device dimensions. Additionally, each measurement is performed over more than thousand structures, being highly representative in a statistical meaning. Finally, independent measurements using electron microscopy corroborate the proposed methodology.

## Introduction

The aggressive roadmap of complementary metal oxide semiconductor (CMOS) transistors technology has been driving for decades the principal advances in nanotechnology fabrication methods, tools and characterization, from which innumerous other science fields benefit today. In the last few years we have experienced a major transition from 2D planar transistors to 3D Fin Field Emission Transistors (FinFET)^1^. Furthermore, devices based on 3D NanoSheet transistors architecture are announced for upcoming sub 5-nm node^2,3^. As a consequence, challenges for the characterization and metrology of increasingly sophisticated materials and processes have to be addressed, now in 3D structures. While traditional 1D characterization techniques such as Secondary Ion Mass Spectrometry (SIMS) are routinely used for fundamental studies/developments, the transposition to intricate topology at such a small dimension and high density is not straightforward^4^. Information on the structural and compositional variations of conformal layer deposition are of high value for the industry, for example in the complex high-k and related oxide layers used for the metal gate^5,6^, or such as doping of 3D transistors for the formation of optimized junctions. More recently, the development of Atom Probe Tomography (APT) offered an inherent 3D technique, but in practice, it revealed the need of considerable further developments from sample preparation to reconstruction algorithms in order to be used as a reliable technique with reasonable throughput^7^. Therefore, at the moment, the task relies fundamentally on Transmission Electron Microscopy (TEM) based techniques. However, this implies into special care on artifacts of sample preparation and, as well as for APT, intrinsic limited statistics. As a complementary technique to address some of these issues, here, we extend the capabilities of Medium Energy Ion Scattering (MEIS) to develop a non-destructive method for the structural characterization of 3D electronic nanostructures. To demonstrate the approach we used an array of Silicon-On-Insulator fins doped with As ions by Plasma Immersion Ion Implantation (PIII). Using a two-fold angular MEIS detection, we separate the signal from backscattered ions that follows an unperturbed flight back to the detector from the ions that cross the fins. While the energy loss of ions provides the information on the chemistry of the different 3D components of the structure, we also show that the periodicity of the fin array generates oscillatory features as a function of the ion energy and the detection angle, allowing the determination of height, width and average interdistance between fins (fin-pitch) i.e. a full compositional and dimensional reconstruction. Each measurement is performed over thousands of fin structures rendering very high statistics. The structure is reconstructed by data simulation using the PowerMEIS code^8^.

## Results

**Figure 1:**
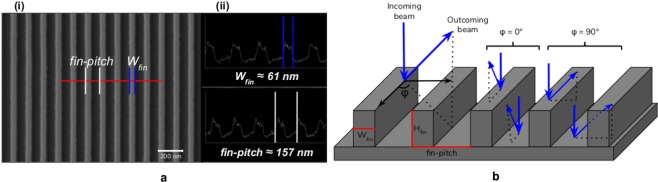
(**a**) Top-view of the fin array obtained by SEM (**i**) and profiles of the fin structures showing the fin width and fin-pitch (**ii**). (**b**) Sample illustration and experimental geometry used for MEIS measurements. The sample was fixed in two different positions which allows the measurement of outgoing ion paths crossing (*φ* = 90°) and along (*φ* = 0°) the fin.

Figure 1(a) shows a top-view of the array of Silicon-On-Insulator fins doped with As ions by PIII (fin array) obtained by Scanning Electron Microscopy (SEM) and profiles of the fin structures displaying the fin width and fin-pitch, respectively. From these images, values of 61 and 127 nm were found for the fin width and fin-pitch. A cross-section sketch to illustrate the sample layout is shown in Fig. 1(b). As can be observed, the sample consists of fin structures parallel to each other. The MEIS technique can obtain structural and compositional information of this fin array with nanometric spatial resolution and high statistics. To that end we consider two geometries where the incident ions have different paths inside the material, as illustrated in the Fig. 1(b). Specifically, two exit trajectories are selected: crossing (*φ* = 90°) and along (*φ* = 0°) the fin. Figure 1(b) also shows the incoming and outgoing paths of the backscattered ion at the top of the fin and at the bottom between the fins for both geometries. Only the outgoing ion path changes from *φ* = 0° to *φ* = 90°, where the *φ* = 90° geometry is sensitive to the structure of the fins. The corresponding outgoing paths have different energy losses, and therefore can be traced out by measuring the energy of the backscattered ions. With an energy resolution of ΔE/E = 4 × 10^−3^ the MEIS technique can resolve the energy loss along and across the fin array allowing for a complete characterization of the fin dimensions and their dopant profile.

**Figure 2:**
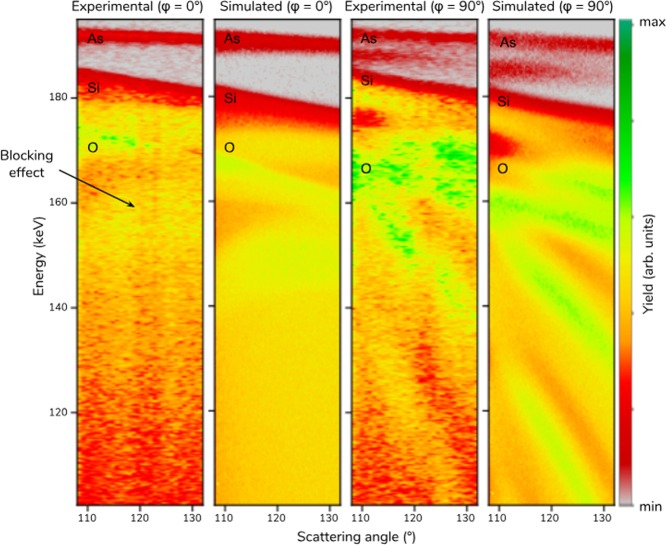
Experimental and simulated map of the proton yield as a function of the backscattered energy and angle (the so-called 2D MEIS spectrum) for 200 keV H^+^ ions at two geometries: along (*φ* = 0°) and crossing (*φ* = 90°) the fins. The color maps represents the backscattered proton yield.

**Figure 3:**
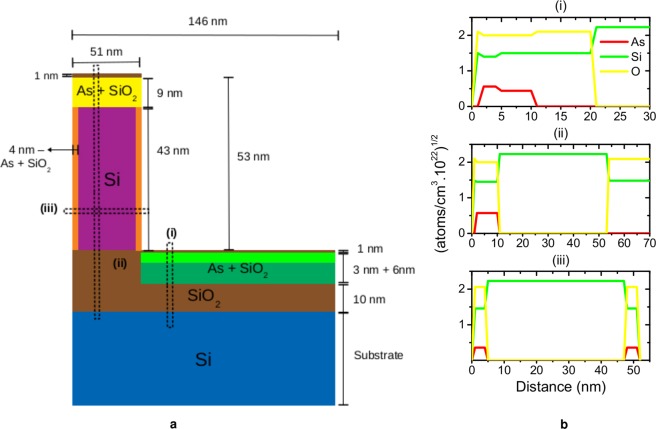
(**a**) Sketch of the fin structure and dopant profiles that best fit the experimental data shown in Fig. 2. (**b**) Depth distribution of As, Si and O for the bottom and top part of the structure as well as perpendicular to the walls.

The MEIS results, namely the maps of 200 keV proton yield as a function of energy and angle of the ions, are shown in Fig. 2. The ion-beam incidence and detection directions are depicted in Fig. 1(b). The corresponding simulations performed by the PowerMEIS code (available online^8,9^) are also shown in Fig. 2. For the simulations, the As implanted in the fin array is described by voxels (a sub-nm regular grid in notational three-dimensional space) according to the model presented in Fig. 3(a) applying periodic conditions. The contributions from protons backscattered from As, Si and O are easily distinguished in Fig. 2. Moreover, the effect of blocking (reduced intensity at certain scattering angles) on the Si signal along a main crystallographic direction can be seen in the experimental 2D spectrum. For elements at the surface of the sample, the signals have different slopes according to the dependence of the kinematic factor on the scattering angle.

The As signal for *φ* = 0° corresponds to the sum of signals from the top of the fin and the bottom between the fins since this geometry does not distinguish both distributions. On the other hand, for *φ* = 90°, the backscattered ions at the bottom between the fins lose more energy by crossing one or more fins before being detected and therefore the As signal is separated in two components (Fig. 2). Thus, the total amount of As in these two regions of the structure can be obtained straightforwardly. The quantification of As on the walls is somehow indirect as its signal is overlapped with other As peaks from top and bottom. It requires a good fitting of the shape of the Si in the energy spectrum in two geometries (across and along the fins). Moreover, for *φ* = 90°, strong oscillatory features are observed in the map of the proton yields as a function of the ion energy and they reveal the FinFET periodic structure. They are nicely reproduced by the simulations. The blocking lines visible in the experimental spectra are not present in the simulations since the PowerMEIS code does not take into account crystalline structures. Channeling effects happen along the way in before the backscattering collision and can be minimized by a slight variation of the incidence angle. Since most of the crystalline part of the FinFETs is buried channeling effects are reduced and smaller than the effects from the periodic structure observed only for *φ* = 90°. Note that for *φ* = 0°, the effects from the periodic fin structure vanish and therefore possible channeling effects can be better differentiated. Channeling and blocking effects could be employed to obtain information about the configuration of As in the Si lattice^10^.

**Figure 4:**
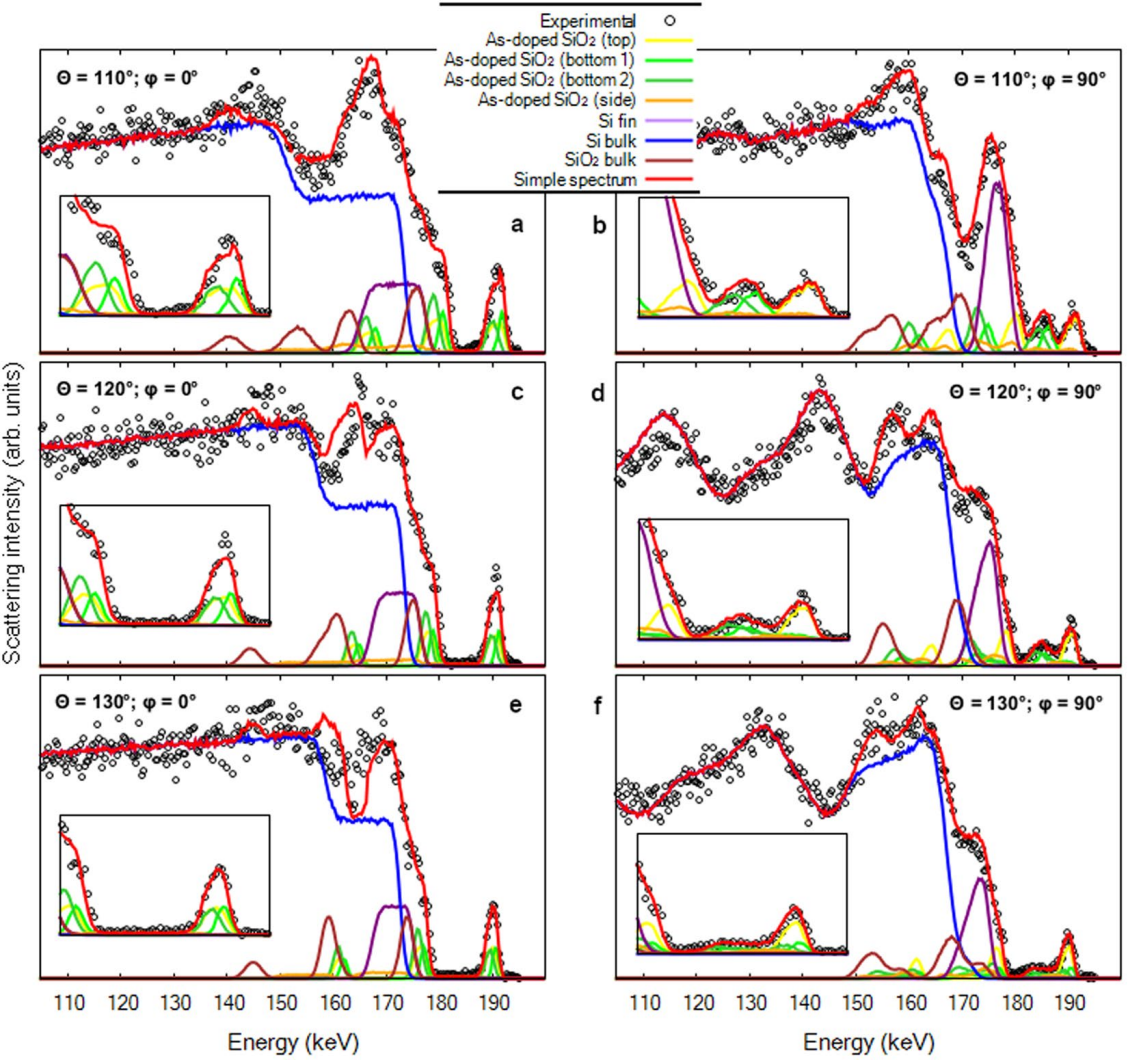
MEIS energy spectra for three scattering angles summed in an interval of ±2°. MEIS simulations for the model described in Fig. 3(a) are displayed for different geometries *φ* = 0° (**a**,**c** and **e**) and *φ* = 90° (**b**,**d** and **f**) and angular regions: 108–112° (**a** and **b**); 118–122° (**c** and **d**); and 128–132° (**e** and **f**). The red lines correspond to the sum of all proton yields. A zoomed-in inset shows the As peak region in detail.

A careful analysis of the experimental map shown in Fig. 2 provides the fin structure dimensions and the full As 3D distribution. This is accomplished by comparing experimental and simulated energy spectra at different angular regions. They are obtained through the projection of the 2D map on the energy axis for a particular angular aperture. Similar maps can be also found in Scattering And Recoil Imaging Spectra (SARIS)^11^ but for other ion energies. In order to improve the counting statistics, several angle bins are summed centered at 110, 120 and 130°. These energy spectra are shown in Fig. 4 by open circles and the corresponding simulations by a red line. PowerMEIS allows the decomposition of the total scattering yield for each element or compound, facilitating their location in the structure. These partial spectra are also shown in Fig. 4 using the same colors as in Fig. 3(a). The inset in these figures show the high energy part of each spectrum zooming the As profile down to the onset of the Si signal from the silicon dioxide (SiO_2_) compound. The shape of this Si signal (see inset) depends strongly on the stoichiometry of the As-doped SiO_2_. The model shown in Fig. 3(a) corresponds to the fin structure and As dopant profiles which best fit the experimental data. The goodness of fit was accessed by employing the reduced *χ*^2^ as the figure-of-merit^12^ (a brief description is given in the Supplementary Information (SI)). The values for the height (H_*fin*_), width (W_*fin*_) and fin-pitch are 53, 51 and 146 nm, respectively. The As, Si and O depth profiles are displayed in Fig. 3(b) and further details of the As depth profiles at the top, wall of the fin structure and bottom (region between the fins) are shown in Table 1. We also observe a diffusion profile of the As located at the bottom.

**Table 1:** PowerMEIS results for composition, thickness, density and dose of As in the fin array depicted in Fig. 3(a).

	Composition	Thickness (nm)	Density (g/cm^3^)	Dose (atoms/cm^2^)
As_top_	As_0.05_(SiO_2_)_0.95_	9.0	2.5	3.4 × 10^15^
As_wall_	As_0.01_(SiO_2_)_0.99_	4.0	2.2	1.0 × 10^15^
As_bottom_ – 1^st^ layer	As_0.05_(SiO_2_)_0.95_	3.0	2.5	1.0 × 10^15^
As_bottom_ – 2^nd^ layer	As_0.03_(SiO_2_)_0.97_	6.0	2.4	1.2 × 10^15^
As_total_	—	—	—	2.1 × 10^15^

Regarding the proton yield from collisions with Si atoms there is a smaller number of oscillations as a function of the ion energy for *φ* = 0° than for *φ* = 90°. Particularly, oscillatory features for *φ* = 90° give information on the fin structure with an estimated spatial resolution of 3 nm, as shown by the *χ*^2^ analysis in the SI. Finally, the overall agreement between simulations and experimental results is remarkable although some deviations are found at energies between 155 and 170 keV for *φ* = 0°, possibly related with channeling effects.

**Figure 5:**
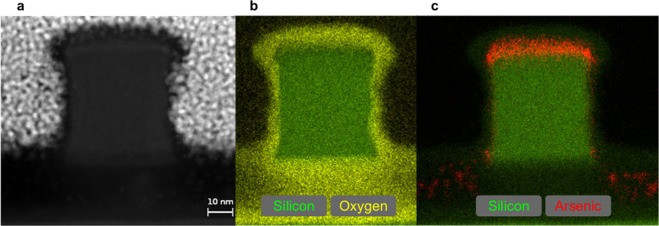
(**a**) HAADF-STEM image of a single fin. (**b**) EDX map for silicon and oxygen. (**c**) EDX map for silicon and arsenic.

The fin-pitch results obtained by SEM and MEIS are in good agreement (difference of 7.2%). A difference of 17.8% is found for W_*fin*_ and can be understood from Scanning Transmission Electron Microscope (STEM) images. A single fin and its corresponding elemental distribution were characterized by the STEM coupled with Energy-Dispersive X-ray spectroscopy (STEM-EDX) technique. Figure 5(a) shows a High-Angle Annular Dark-Field (HAADF) STEM image of a single fin, while Fig. 5(b,c) show the STEM-EDX images for the silicon/oxygen and silicon/arsenic elemental concentrations, respectively. As a matter of fact the shape of the fins is not a rectangular parallelepiped as assumed during MEIS simulations. The top is wider than the middle part of the fin as a consequence of the etching process used to design such structures. However, the difference between the top and the middle of the fin is approximately 4 nm, which is close to our spatial resolution of 3 nm over all FinFET dimensions. Therefore we cannot distinguish between the simplified model proposed and the FinFET structure showed by the STEM image. In this way, the value of W_*fin*_ corresponds to the middle of the fin structure in the dimensional analysis through the STEM images. As the top of the fin is wider than the middle part, the SEM images overestimate the W_*fin*_ value and therefore this dimension is larger in comparison to MEIS analyses.

From STEM images, the values of H_*fin*_, W_*fin*_ and fin-pitch are 55, 47 and 156 nm, respectively. The largest discrepancy between the results obtained by MEIS and STEM occurs for W_*fin*_ and corresponds to 8.1%. STEM-EDX also shows As diffusion in the bottom part between fins and the presence of an uniform As layer at the top and the wall of the fin (see Fig. 5(c)). These observations clearly corroborate the MEIS-based methodology proposed here. The elemental depth profile obtained by STEM-EDX is shown in the SI.

## Discussion

A conventional approach to determine the dopant distribution in FinFET devices is the SIMS technique^13,14^. In order to characterize 3D structures, it was necessary to develop new methodologies since traditional SIMS gives only the one-dimensional (1D) depth profile. Examples of such approaches are the array profiling^15^, 1.5D-SIMS^4,16^ and more recently the self-focusing SIMS (SF-SIMS)^17^. These new methodologies allow measurements on confined volumes without having to push the beam to nm-scale dimensions^4^. However, with the exception of SF-SIMS, not only the geometry of the structure must be previously known (e.g. using STEM), but also the structure needs to be periodic in a highly reproducible manner^13^. APT and Scanning Spreading Resistance Microscopy (SSRM) were also used to profile dopant and carrier distributions in FinFET-based devices with sub-nanometer resolution. Nevertheless, these techniques still have practical drawbacks as the sample preparation, the analysis conditions, and 3D-data reconstruction (local magnification effects)^4,7,18^.

On the other hand, the MEIS technique is able to provide dopant profiles for different parts of the fin structure and the FinFET dimensions simultaneously. Nowadays, it is the only technique capable of quantifying the chemical and structural properties of FinFET devices statistically. MEIS results are in fact an average over thousands of FinFET structures. It is pointed out that differently from SIMS, MEIS relies on stopping powers and cross-sections. The first implies in a depth uncertainty of the order of 4% according to the accuracy of the present stopping-power tables. The detection sensitivity determined by cross-sections is generally poorer than detecting directly secondary ions as in SIMS. Concerning the As implanted, we estimate a total dose of 2.1 × 10^15^ atoms/cm^2^ (see Table 1), which is about half the nominal dose (5.0 × 10^15^ atoms/cm^2^). This dose difference can be understood from the fact that PIII process does not use a mass selector, therefore, a mix of compounds and neutral species are deposited on the substrate surface^19^. The As distribution obtained from MEIS and STEM-EDX agree with each other. Our results show an uniform As profile at the top and a diffusion-like one at the bottom. This difference probably comes from the SiO_2_ layers at the top and bottom parts before the As implantation. Different from the top, which had only the native oxide, the bottom part of the FinFET had a much thicker SiO_2_ layer due to the SOI structure used to create the fins by chemical etching. Investigations in planar systems^20,21^ show that the As implanted in Si through a narrow native oxide, can interact with the oxygen from the atmosphere resulting in a SiO_2_ layer of approximately 10 nm in which the As is distributed almost homogeneously. Therefore, the As profile at top should be more uniform than at bottom where the As was implanted only in the SiO_2_ layer. The profiles for MEIS and STEM-EDX are shown in Figs 3 and S9 (SI), respectively. The fin array dimensions obtained by MEIS, SEM and STEM are summarized in the SI.

The disagreement on the value of W_*fin*_ obtained via MEIS and STEM with that obtained via SEM can be explained by the fact that the top part of the fin is wider than the middle one due the etching process used to design the fin array. For the H_*fin*_ and fin-pitch, the comparison between MEIS and STEM shows a difference of 2.5% and 6.6%, respectively. In addition, the manufacturing process of these structures cannot guarantee that all fins have the same dimensions. For this reason, the statistical analysis provided solely by the MEIS technique becomes advantageous.

The studied array of Silicon-On-Insulator fins doped with As is not intended to be an operational device since no cleaning and annealing processes were performed in the array. However, the sample has all the dimensional characteristics to allow for a demonstration of MEIS capability to quantify the distribution of dopants over the 3D nanostructure (close to that used in real FinFET devices) and its main dimensions (H_*fin*_, W_*fin*_ and fin-pitch). In summary we demonstrated the capability of the MEIS technique to characterize 3D FinFET electronic devices. Besides the fin parameters (H_*fin*_, W_*fin*_ and fin-pitch), which are responsible for strong oscillatory features observed in the 1D and 2D MEIS spectra, the determination of the As profile along the top, bottom and wall of the fins is the most promising result. It opens new perspectives for the use of ion scattering in microelectronic technology.

## Methods

MEIS measurements were carried out at the Ion Implantation Laboratory of the Institute of Physics (Federal University of Rio Grande do Sul, Brazil). A 500 kV electrostatic accelerator provided an incident beam of H^+^ with nominal energy of 200 keV. Fin structures were mounted in a triple-axis goniometer that allowed to perform measurements with outgoing ion path along (*φ* = 0°) and crossing (*φ* = 90°) the fin structures as shown in Fig. 1(b). The sample was kept inside the analysis chamber under a pressure of about 10^−7^ mbar. Typical beam current was smaller than 15 nA (accumulated charge did not exceed 0.02 C/cm^2^). The detection system consists of a Toroidal Electrostatic Analyzer (TEA) that collect the backscattered H^+^ ions from the collision between the incident beam and the sample. At the top end of the TEA a set of two micro-channel plates coupled to a position-sensitive detector allows the determination of the scattering energy and angle for each impinging ion^22,23^. This detection system was centered at 120° with respect to the incident beam. The TEA angular aperture is 24° and each angle bin corresponds to 0.08°. The overall energy resolution of the system, considering an incident beam of 100 keV H^+^, is 450 eV. The PowerMEIS code (available online) was used to analyze the experimental results^8,9^. Data were selected in three different angular regions: 108–112, 118–122 and 128–132°. The reduced chi-square^12^ was used as a figure-of-merit.

SEM images were acquired with a Carl Zeiss Auriga FIB/FEG-SEM microscope equipped with in-lens SE detector. The overall resolution for 15 keV electrons is 1 nm. HAADF images were acquired using STEM in a Cs-corrected Titan 80/300 FEI/Thermo Fisher Scientific microscope operated at 300 kV. The typical lateral resolution was greater than 0.1 nm. Space-resolved elemental analyses were performed via EDX in STEM mode. Drift-corrected elemental mapping was carried out in a Titan Cubed Themis FEI/Thermo Fisher Scientific using the Super-X quad EDX detector. The cross-sectional STEM samples were previously prepared using Focused Ion Beam (FIB) protocols for lamella preparation with a FEI Helios NanoLab DualBeam.

## Supplementary information

Supplementary information without highlight
