# Supplementary material for: Unraveling structural and compositional information in 3D FinFET electronic devices
Source: Sci Rep. 2019 Aug 12;9:11629. doi: 10.1038/s41598-019-48117-0 (PMC6691105; doi:10.1038/s41598-019-48117-0)
Supplement: Supplementary file 1 — Supplementary information without highlight [file 41598_2019_48117_MOESM1_ESM.pdf]

Unraveling structural and compositional information in 3D FinFET electronic devices

Henrique Trombini[1,*], Gabriel Guterres Marmitt[1], Igor Alencar[1], Daniel Lorscheitter Baptista[1], Shay Reboh[2], Frédéric Mazen[2], Rafael Bortolin Pinheiro[2], Dario Ferreira Sanchez[3], Carlos Alberto Senna[4], Bráulio Soares Archanjo[4], Carlos Alberto Achete[4], and Pedro Luis Grande[1]

[1]Ion Implantation Laboratory, Institute of Physics, Federal University of Rio Grande do Sul, CEP 91501-970 Porto Alegre, Brazil
[2]CEA-LETI, MINATEC Campus, F-38054 Grenoble, France
[3]Paul Scherrer Instituit, 5232 Villigen, Switzerland
[4]National Institute of Metrology, Quality and Technology, CEP 25250-020 Duque de Caxias, Brazil
[*]henrique.trombini@ufrgs.br

ABSTRACT

Non-planar Fin Field Effect Transistors (FinFET) are already present in modern devices. The evolution from the well-established 2D planar technology to the design of 3D nanostructures rose new fabrication processes, but a technique capable of full characterization, particularly their dopant distribution, in a representative (high statistics) way is still lacking. Here we propose a methodology based on Medium Energy Ion Scattering (MEIS) to address this query, allowing structural and compositional quantification of advanced 3D FinFET devices with nanometer spatial resolution. When ions are backscattered, their energy losses unfold the chemistry of the different 3D compounds present in the structure. The FinFET periodicity generates oscillatory features as a function of backscattered ion energy and, in fact, these features allow a complete description of the device dimensions. Additionally, each measurement is performed over more than thousand structures, being highly representative in a statistical meaning. Finally, independent measurements using electron microscopy corroborate the proposed methodology.

Supplementary Information

Sample preparation

Structures were fabricated in state of the art 300 mm semiconductor facilities. Silicon-On-Insulator substrate with initial 14 nm and 25 nm thicknesses of Si-Top and buried oxide respectively follows an epitaxy of 46 nm of Si to obtain a total Si layer of 60 nm. The top-Si layer was patterned using lithography to obtain fin-like structures of 60 nm height running to 1.2 mm long. The array of fins extends along 10 mm with a targeted fin-pitch of 160 nm. The sample layout is shown in Fig. S1.

Analysis of the distance traveled by the ion in fin array

In order to analyze the major differences in the distances traveled by the ion in the material for different geometries with $\varphi = 0°$ and 90°, we constructed a new structure using only Si atoms based on previous MEIS results (Fig. 3 (a)). This structure is repeated ten times. The total distance traveled by the ion within the sample was obtained by the PowerMEIS code. In these simulations, we consider an incident beam of 200 keV H^+ that impinges normal to the surface and a backscattering angle centered at 120° with an angular aperture of 4°. The Fig. S2 shows the distance traveled in the material relative to depth and horizontal position for the two experimental geometries. Let us consider the depth dependence of the distance traveled by the ion in the fin. For the $\varphi = 0°$ case, the ions travel the same distance and have the same displacement, regardless of their horizontal position. However, this does not happen for the $\varphi = 90°$ case, as shown in Fig. S2 (b). In this situation, the ion will travel different distances in the fin for the same depth, depending on its horizontal position. This is because the probability of the ion crossing one or more fin structures during its outgoing path depends on its horizontal position. The same reasoning is valid for the bottom part between the fins. It is this pattern that produces oscillatory features in the MEIS spectra[1,2].

Figure S3 shows the distribution of the number of voxels as a function of the distance traveled by the ion in the material for the $\varphi = 90°$ geometry (histogram with red area). Using the appropriate stopping power for the ion-sample configuration, this

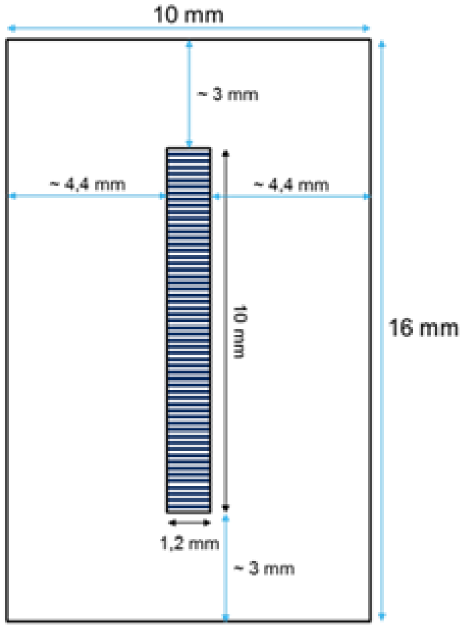

Figure S1. Layout of the sample used.

distance is converted to energy. As can be observed in this figure, a good agreement with the results of direct simulations (gray line) is obtained concerning the period and shape of the oscillatory features of this histogram. As expected, for φ = 0° (blue line) the MEIS spectrum does not present any oscillatory features, varying only with the cross-section dependence.

Density and dose calculations

The density of each layer containing a compound with a combination between As and SiO_2 was calculated considering the volume and stoichiometry of each element in the compound. The volume was calculated according to the Equation S1:

$$V_i = \frac{m_i}{N_a \rho_i} \tag{S1}$$

where V_i is the volume of each element present in the compound, N_a is Avogadro's number, m_i and ρ_i are the atomic mass and the density of this element, respectively. According to the Equation S1, the volumes for As and Si are $V_{As} = 2.16 \times 10^{-23}$ cm^3 and $V_{Si} = 1.99 \times 10^{-23}$ cm^3. For the oxygen, the volume was calculated by $(V_{SiO_2} - V_{Si})/2$ and corresponds to $V_O = 1.26 \times 10^{-23}$ cm^3. Once the volume occupied by each element has been calculated we can determine the density according to Equation S2:

$$\rho_{region} = \frac{\sum_{i=1}^{n} x_i m_i}{\sum_{i=1}^{n} x_i V_i} \tag{S2}$$

where ρ_{region} is the density of each region containing the As-doped SiO_2 compound and x_i is the stoichiometry of the i-th element. Equation S2 is used to calculate the density at the top, wall and bottom (first and second layers) compounds. For example, the density at the top is given by:

$$\rho_{top} = \frac{0.31 m_{\mathrm{Si}} + 0.63 m_{\mathrm{O}} + 0.05 m_{\mathrm{As}}}{0.31 V_{\mathrm{Si}} + 0.63 V_{\mathrm{O}} + 0.05 V_{\mathrm{As}}} \tag{S3}$$

All results are presented in Table 1.

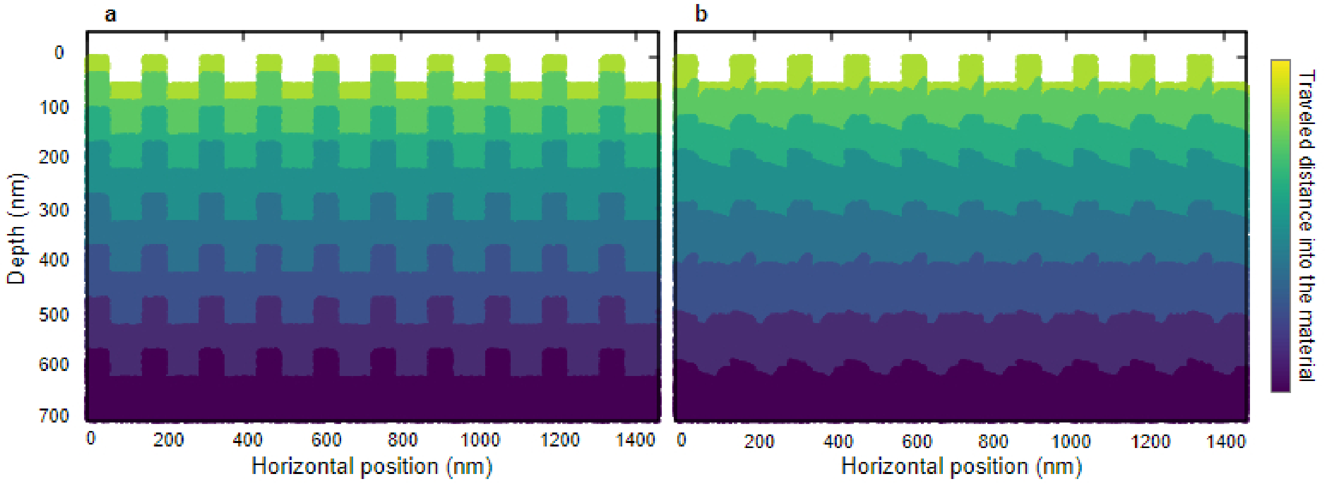

Figure S2. Distance traveled in the material as a function of depth and the horizontal position for the experimental geometries with (**a**) $\varphi = 0°$ and (**b**) $\varphi = 90°$. These simulations were performed considering an incident beam of 200 keV H^+ impinging normal to the surface and a backscattering angle centered in 120°.

In order to determine the implanted dose, it is necessary to calculate the number of As atoms incorporated in each region first. For this calculation, we used Equation S4:

$$N_{atoms_{\mathrm{As}}} = \frac{x_{\mathrm{As}}}{\sum_{i=1}^{n} x_i V_i} V_{\mathrm{region}} \tag{S4}$$

where $N_{\mathrm{atoms_{As}}}$ is the number of As atoms in the region, x_{As} is the As stoichiometry and V_{region} is the volume of the region. In this way, it is possible to obtained the dose according to Equation S5:

$$Dose = \frac{N_{atoms_{\mathrm{As}}}}{A} \tag{S5}$$

where A is area of the region. In this work, we calculate the dose over each region (top, wall and bottom (first and second layers)) as shown in Table 1. Also, we calculate an average dose, considering the total number of As atoms implanted over the surface (top, wall, and bottom) of the fin array.

PowerMEIS code

PowerMEIS is a Monte Carlo program that performs simulations of the interactions of ions[3] and electrons[4] with matter. The sample is described by voxels organized in a matricial format, which may represent any complex structure with unlimited number of compounds[3]. Figure S4 shows a sketch of the matrix constructed for the fin structure.

The PowerMEIS code determines the incoming and outgoing ion paths by numerical integration of a three-dimensional space from the incident and scattering angles. The simulated spectrum is obtained by the integration of Equation S6 over all sample volume, adding the contribution of all elements.

$$dH_{ij}(E) = N_{ij} Q \sigma_i(E_1, \theta) F(E - E_{out}) F^{+}(E) dE dV \tag{S6}$$

with $E_1 = E_0 - \Delta E_{in}$ and $E_{out} = K_i(\Theta)E_1 - \Delta E_{out}$, where E_0, E_1 and E_{out} are the incident energy, the energy just before the backscattering, and the detected energy, respectively. The $K_i(\Theta)$ is the kinematic factor and ΔE_{in}, ΔE_{out} are the energy losses along the incoming, outgoing ion paths. We calculate the energy losses from the stopping power SRIM library[5]. In the Equation S6, N_{ij} is the atomic fraction of the i-th element in the j-th position, Q the fluence of incident ions, Ω the detector solid angle, $\sigma_i(E_1, \theta)$ the differential scattering cross section for the i-th element, $F(E - E_{out})$ is the energy loss distribution and $F^{+}(E)$ is the neutralization probability correction.

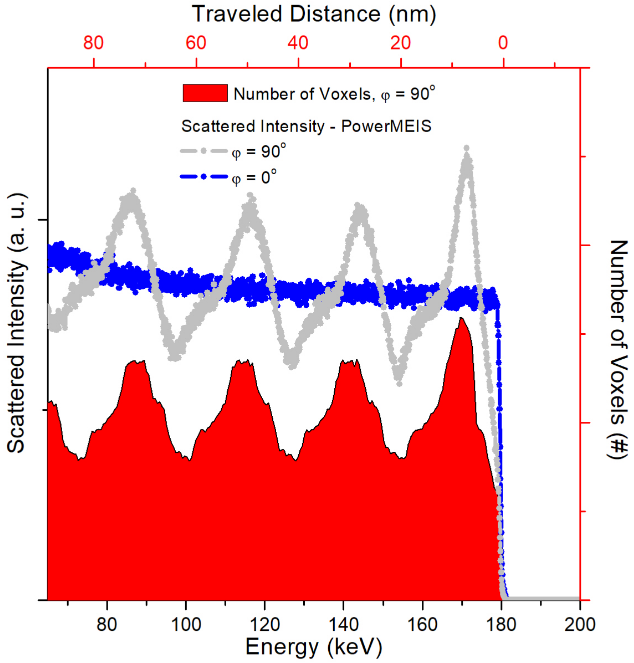

Figure S3. The distribution of number of voxel for a given distance traveled by the ion in the material (red area-filled curve) and MEIS energy spectrum considering $\varphi = 0°$ (blue line) and $\varphi = 90°$ (gray line).

Our simulations have considered the $\sigma_i(E_1, \theta)$ obtained by solving the orbit equation using the Ziegler-Biersack-Littmark interatomic potential[6]. The neutralization $F^+(E)$ is extracted from the Marion and Young data[7]. The energy loss distribution occurs due to the fluctuations in the interaction with target atoms and the detection resolution. For Rutherford backscattering, $F(E - E_{out})$ can be written as a Gaussian function because the detection system cannot resolve the large number of inelastic interactions. On the other hand, these interactions need be taken in account in the MEIS technique. In this way, $F(E - E_{out})$ was written as a Exponential Modified Gaussian (EMG) distribution. The EMG is an analytic formula obtained by the convolution of a Gaussian distribution with an exponential distribution that represents the inelastic energy loss due to ionization and excitation of the backscattered ion[8].

χ^2 analysis

The chi-square is used as a figure-of-merit for the evaluation of goodness of fit for MEIS spectra. In addition, it provides the spatial resolution for each FinFET dimension (chi-square variations larger than 10%). In this work, we used the reduced chi-square given by the Equation S7[9]:

$$\chi^2 = \frac{1}{N}\sum_{i=1}^{N}\left\{\frac{[I_{exp} - I_{sim} + min(I_{exp}, 1)]^2}{I_{exp} + 1}\right\} \tag{S7}$$

where N is the total number of data points, I_{exp} and I_{sim} represent the proton yield in the experimental and simulated spectra, respectively. The $min(I_{exp}, 1)$ factor is used to take into account noises in the experimental data. Figure S5 shows the chi-square results. For each simulation, all dimensions (H_{fin}, fin-pitch or W_{fin}) are fixed except one. In each plot, the chi-square is displayed as a function of this varying dimension for two geometries $\varphi = 0°$ and $\varphi = 90°$ and three scattering angles centered at 110°, 120° and 130°. The $\varphi = 0°$ geometry does not give much information, whereas a clear and narrow minimum is found for $\varphi = 90°$. H_{fin} is determined at small scattering angles (110°) for both geometries. The shape of the spectra for the energies between 155 and 170 keV is crucial for the chi-square value. Our results indicate an uncertainty of 3 nm for H_{fin}. For the determination of fin-pitch and W_{fin} the variations in chi-square for $\varphi = 90°$ are used. In this geometry, any variation in the fin-pitch and width of the fin drastically changes the measured backscattering energy. These results show a sensitivity of about 3 nm for the fin-pitch and W_{fin} dimensions.

The χ^2 analysis shows that small variations in H_{fin}, W_{fin} and fin-pitch increase the discrepancies between the experimental and simulated data. These variations depend strongly on the scattering angle and the irradiation geometry. Figure S6 shows the comparison between the experimental data for backscattering angle 120° and $\varphi = 90°$ with the simulation for the best model

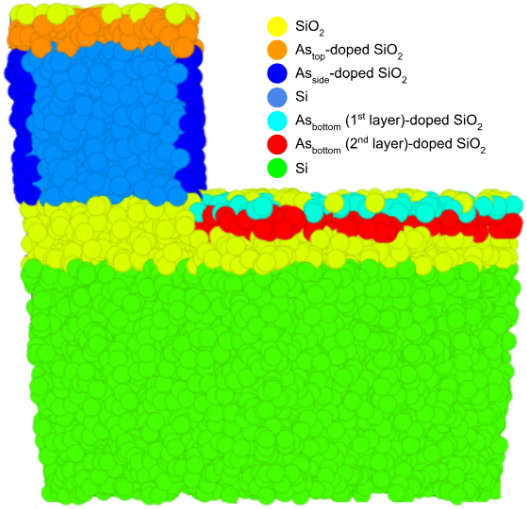

Figure S4. Sketch describing the corresponding matrix constructed for the FinFET structure.

and including a variation of ±3 nm in H_{fin}, W_{fin} and fin-pitch. As presented, the MEIS technique is very sensitive to FinFET size variations. Figure S7 shows the energy spectrum for the backscattering angle 120° and φ = 90° for a FinFET with the following dimensions: (a) H_{fin} = 60 nm, W_{fin} = 20 nm and fin-pitch = 60 nm; (b) H_{fin} = 60 nm, W_{fin} = 40 nm and fin-pitch = 90 nm and (c) H_{fin} = 80 nm, W_{fin} = 140 nm and fin-pitch = 300 nm. For all simulations the structure is composed only of Si.

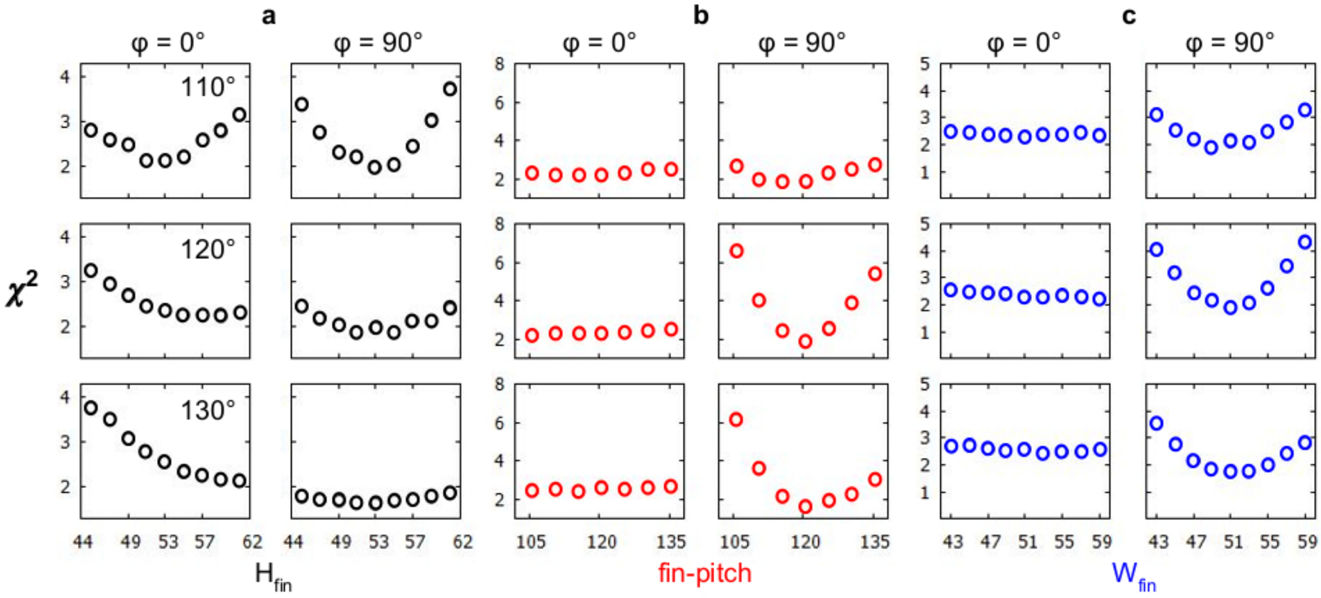

Figure S5. Chi-square analysis over the (**a**) H_{fin}, (**b**) fin-pitch and, (**c**) W_{fin} dimensions.

Experimental details

The analysis was performed in approximately 4 hours. Typical beam current was smaller than 15 nA (accumulated charge did not exceed 0.02 C/cm^2). During the measurement, we change the position of the beam (0.5 × 2 mm^2) every hour (≤5 mC/cm^2 per spot). We have used H^+ as an incident beam which causes much less damage compared to He^+ beams[10]. An additional test

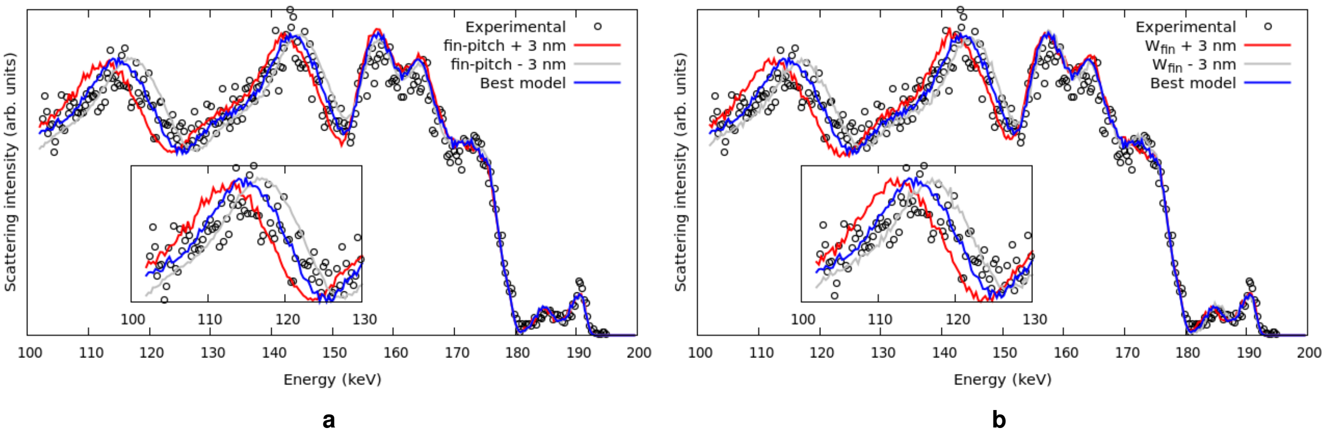

Figure S6. Effect of different dimensions (±3 nm) on the MEIS energy loss spectrum for 200 keV H^+, backscattering angle 120° and φ = 90° on the following dimensions (**a**) fin-pitch and (**b**) W_{fin}.

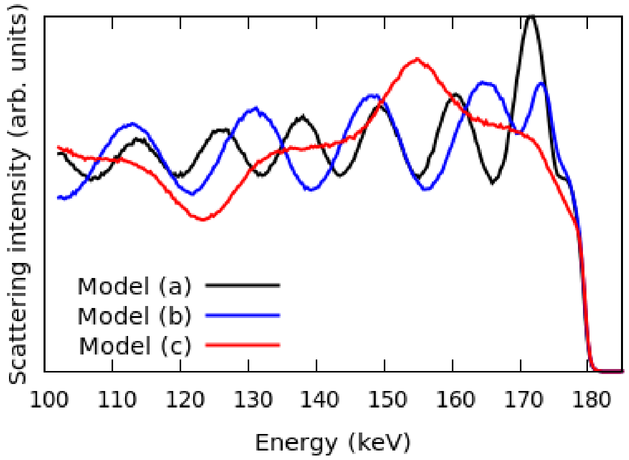

Figure S7. Variations in the energy spectrum for the backscattering angle 120° and φ = 90° for a FinFET composed of Si with dimensions (a) H_{fin} = 60 nm, W_{fin} = 20 nm and fin-pitch = 60 nm; (b) H_{fin} = 60 nm, W_{fin} = 40 nm and fin-pitch = 90 nm and (c) H_{fin} = 80 nm, W_{fin} = 140 nm and fin-pitch = 300 nm.

with two different scan measurements (2 hours each) at the same spot was done and no substantial difference was observed between them, as shown in Figure S8.

STEM-EDX analysis

The As dopant profiles obtained by MEIS and STEM-EDX are in good agreement, as shown in Figs. S9 and 3. The As dopant profile obtained by STEM-EDX is shown for three different regions: (a) horizontal cut over the fin structure, (b) vertical cut over the fin structure and, (c) vertical cut between fins. The first region presents an uniform dopant distribution with low concentration. In this case, the symmetry in the As signals at both sides of the walls depends on the region selected. The second region exhibits an uniform As distribution with a high concentration when compared with the As distributions at the wall and the bottom. As the MEIS results showed, the As distribution is not uniform in the region between fins. The dopant profile of the third region confirms the necessity of a diffusion layer of As. In this case, the As implanted is more concentrated in the first layer than in the second one. Another important agreement between MEIS and STEM-EDX results concerns the thickness of these layers. The As at the fin wall, top and bottom are distributed within a layer of 5, 9 and 10 nm, respectively. At the bottom case, there are two layers, the first with 3 nm and the second with 7 nm.

The fin array dimensions obtained by MEIS, SEM and STEM are summarized in Table S1.

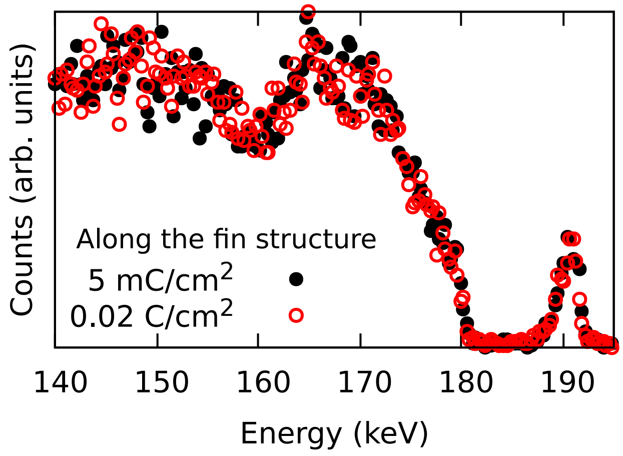

a

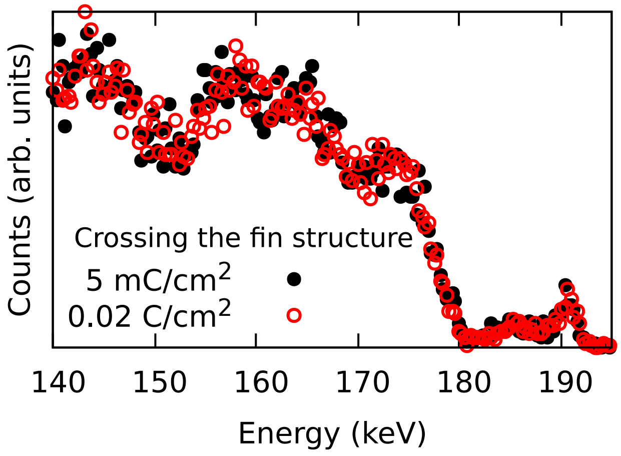

b

Figure S8. Two scan measurements at the same spot (accumulated charge did not exceed 0.01 C/cm^2 per scan) for the two experimental geometries: **a** along ($\varphi = 0°$) and **b** crossing ($\varphi = 90°$) the fin, indicating no substantial difference between them.

Dimensions (nm)	MEIS (I)	SEM (II)	STEM (III)	I-II (%)	I-III (%)	II-III (%)
H_{fin}	53	-	55	-	2.5	-
W_{fin}	51	61	47	17.8	8.1	25.9
fin-pitch	146	157	156	7.2	6.6	0.6

Table S1. Comparison between MEIS (I), SEM (II) and STEM (III) techniques for the fin array dimensions and the relative difference between them.

The depth distribution of As, Si and O (logarithmic scale) for the bottom and top part of the structure as well as perpendicular to the walls is shown in the Fig. S10.

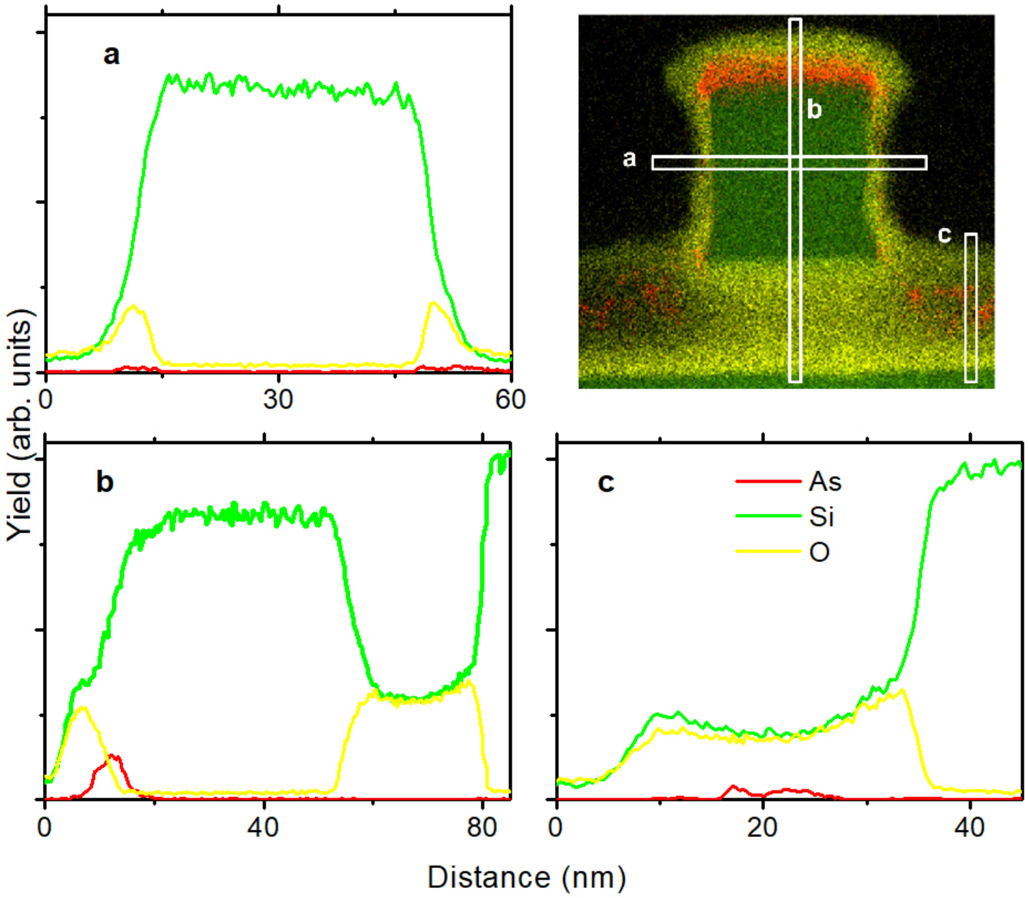

Figure S9. Elemental profile for the arsenic, silicon and oxygen considering the regions **a**, **b** and **c** illustrated in the STEM-EDX image.

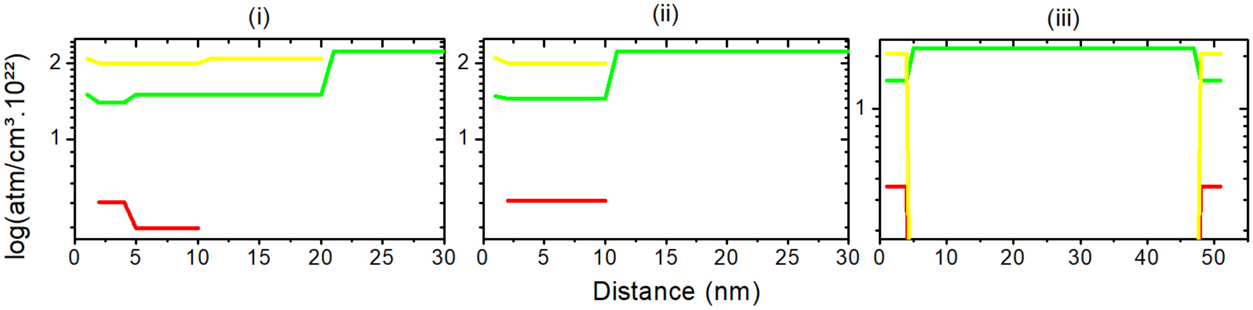

Figure S10. Depth distribution of As, Si and O (logarithmic scale) for the bottom (i) and top (ii) part of the structure as well as perpendicular to the walls (iii).

References

1. Behrisch, R. *et al.* Influence of surface roughness on measuring depth profiles and the total amount of implanted ions by RBS and ERDA. *Nucl. Instrum. Methods Phys. Res. B* **136**, 628–632 (1998).
2. Edge, R. D. & Bill, U. Surface topology using Rutherford backscattering. In *Ion Beam Analysis*, 157–162 (Elsevier, 1980).
3. Sortica, M. A. *et al.* Characterization of nanoparticles through medium-energy ion scattering. *J. Appl. Phys.* **106**, 114320 (2009).
4. Vos, M., Grande, P. L. & Marmitt, G. G. The influence of shallow core levels on the shape of REELS spectra. *J. Electron Spectrosc. Relat. Phenom.* **229**, 42–46 (2018).
5. Ziegler, J. F. The stopping and range of ions in matter. http://www.srim.org/.
6. Ziegler, J. F. & Biersack, J. P. The stopping and range of ions in matter. In *Treatise on Heavy-Ion Science*, 93–129 (Springer, 1985).
7. Marion, J. B. & Young, F. C. *Nuclear Reaction Analysis, Graphs and Tables* (North Holland Publishing Co, Amsterdam, 1968).
8. Pezzi, R. P. *et al.* Analytical energy loss distribution for accurate high resolution depth profiling using medium energy ion scattering. *Appl. Phys. Lett.* **92**, 164102 (2008).
9. Mighell, K. J. Parameter estimation in astronomy with Poisson-distributed data. I. The χ^2_γ statistic. *Astrophys. J.* **518**, 380 (1999).
10. Buck, T. M. & Wheatley, G. H. Studies of solid surfaces with 100 keV $^4He^+$ and H^+ ion beams. *Surf. Sci.* **33**, 35–55 (1972).
